# Alzheimer's disease and neurodegenerative plasma biomarkers predict altered sleep neurophysiology in older adults with subjective and objective cognitive impairment

**DOI:** 10.1002/alz70856_101696

**Published:** 2025-12-25

**Authors:** Aaron Kin Fu Lam, Johannes C. Michaelian, Rachael Yu, Nathan Cross, Angela D'Rozario, Sharon L. Naismith

**Affiliations:** ^1^ Woolcock Institute of Medical Research, Macquarie Park, NSW, Australia; ^2^ The University of Sydney, Camperdown, NSW, Australia; ^3^ Healthy Brain Ageing Program, Brain and Mind Centre, University of Sydney, Camperdown, NSW, Australia; ^4^ Healthy Brain Ageing Program, Brain and Mind Centre, School of Psychology, Faculty of Science, University of Sydney, Camperdown, NSW, Australia; ^5^ School of Psychology, Faculty of Science, University of Sydney, Sydney, NSW, Australia; ^6^ Healthy Brain Ageing Program, Brain and Mind Centre, University of Sydney, Sydney, Australia; ^7^ School of Psychology, Faculty of Science, University of Sydney, Sydney, Australia; ^8^ Woolcock Institute of Medical Research, Macqaurie Park, NSW, Australia; ^9^ Healthy Brain Ageing Program, University of Sydney, Sydney, Australia; ^10^ Charles Perkins Centre, University of Sydney, Sydney, NSW, Australia

## Abstract

**Background:**

Emerging evidence suggests altered sleep neurophysiology may be an early marker of brain pathology. However, no studies have examined links between sleep neurophysiology and blood‐based neurodegeneration biomarkers. We aimed to determine if blood‐based Alzheimer's and neurodegeneration biomarkers are associated with sleep neurophysiology in older adults with subjective and objective cognitive impairment.

**Method:**

We recruited adults aged 50+ with cognitive or mood concerns from a memory clinic. Cognitive status was classified as mild cognitive impairment (MCI) or subjective cognitive impairment (SCI) using neuropsychological test criteria. MCI required scores ≥1.5 SD below normative means on one or more tests, while SCI indicated no objective deficits. Participants completed a fasting blood test and overnight polysomnography. Plasma biomarkers (Aβ42/40, GFAP, NFL, pTau181) were measured on the Quanterix SIMOA HD‐X platform using commercially available kits. Sleep measures included spindle density, relative theta/delta power, and REM slowing. Spindles were detected via a validated algorithm (C3), while power and REM slowing used Fz electrode data. Linear regressions assessed whether biomarkers predicted sleep neurophysiology metrics.

**Result:**

Our sample included 116 participants (mean age=68.3 (SD=8.1), *n* = 40 males (34.5%)), with a mean Mini‐Mental State Examination of 28.3 (SD=2.5). Of these, 56 (48.3%) had MCI, and 60 (51.7%) had SCI. Spindle density and relative delta power were higher in SCI than MCI (*p* <0.05), whereas relative theta was lower in SCI (*p* <0.05). No group differences were found in REM slowing (*p* >0.05). As expected, Aβ42/40 was lower in MCI than SCI (*p* <0.05), while GFAP, NFL, and pTau181 were higher in MCI (*p* <0.05). Regression analyses showed Aβ42/40 was associated with spindle density (R^2^=0.03, t=‐2.2, *p* <0.05) and REM slowing (R^2^=0.03, t=2.1, *p* <0.05). GFAP (R^2^=0.05, t=‐2.5, *p* <0.05) was significantly linked to spindle density. After adjusting for clinical status and age, only REM slowing remained significant (*p* <0.05).

**Conclusion:**

The findings suggest REM slowing is a robust marker linked to plasma Aβ42/40, highlighting its potential as an early indicator of neuropathological changes. Aligned with prior research, REM slowing may reflect pathological changes driven by amyloid accumulation, contributing to disrupted cholinergic signalling. Longitudinal studies are needed to determine the causal relationship between sleep neurophysiology and plasma biomarkers of AD and neurodegeneration.

